# Is Deterioration of Surface Properties of Resin Composites Affected by Filler Size?

**DOI:** 10.1155/2020/2875262

**Published:** 2020-01-04

**Authors:** H. Elbishari, N. Silikas, J. D. Satterthwaite

**Affiliations:** ^1^Restorative Department, College of Dentistry, Ajman University, Ajman, UAE; ^2^Division of Dentistry, Faculty of Biology Medicine and Health, The University of Manchester, Manchester, UK

## Abstract

**Purpose:**

To investigate the effect of filler size on surface gloss and roughness of resin composites using a glossmeter and 3D noncontact surface topography, respectively, before and after tooth-brushing abrasion.

**Materials and Methods:**

Seven model resin composites and one commercial were tested in the study. All materials were first polished, and then the surface gloss and 2D and 3D roughness parameters were recorded. Materials are then subjected to abrasion in a tooth-brushing simulator. Roughness parameters were recorded after 10,000 cycles, and after 20,000 cycles, both roughness and gloss were recorded. One-way ANOVA and Bonferroni post hoc test (*p* < 0.05) were used to analyze data.

**Conclusion:**

Filler size is strongly correlated to gloss and surface roughness retention.

## 1. Introduction

Resin composites have been increasingly used in restorative dentistry for more than half a century [1] and are used routinely for restorations in the anterior and posterior teeth. They feature a wide range of aesthetic and mechanical properties making them the most widely used tooth-coloured material for restoration of teeth [2]. However, they still exhibit drawbacks in terms of polymerization shrinkage, wear and loss of aesthetics upon use. Filler particle technology is an important factor influencing both physical [3, 4] and mechanical properties [5]. Improvements of surface smoothness and gloss retention can be achieved by reducing the filler size [6, 7].

Resin composites containing nanosized fillers can offer better aesthetics [8] and better wear resistance [9]. Finishing and polishing are important not only for aesthetic reasons but also for the longevity of restoration [10] and the gingival and periodontal health. This is because the surface texture of resin composites has an influence on plaque accumulation, which may lead to gingival and periodontal inflammation and also discoloration of restorations [11]. There is a direct correlation between surface roughness and plaque accumulation; as surface roughness increases, so does the deposition of plaque [12]. Previous in vitro studies showed that mean roughness (Ra) above the 0.2 *μ*m threshold was related to a substantial increase in bacteria retention on the surface of the restoration [12]. There are several studies measuring surface roughness of resin composites. However, there are limited studies exploring the surface roughness using 3D noncontact method.

The objective of the present study was to assess the effect of different filler sizes on the gloss and surface roughness (2D and 3D measurements) of resin composites before and after tooth-brushing abrasion. A series of model composites with varying filler size and distribution were examined. Also, a noncontact 3D method to evaluate surface roughness was employed. The following null hypotheses were formulated:

- Filler size has no effect on the gloss retention of resin composite materials
- Filler size has no effect on surface roughness of resin composite materials

## 2. Materials and Methods

Seven model resin composites (Ivoclar Vivadent, Schaan, and Liechtenstein) and one commercial resin composite (Tetric Ceram, Ivoclar Vivadent, Schaan, and Liechtenstein) were investigated in this study. All resin composites (model and commercial) were visible light-cured composites containing the same resin matrix which was a combination of Bis-GMA, UDMA, and TEGDMA, with camphoroquinone. All model composites had a dispersed phase with the same volume fraction (56.7%), which was treated with a silane coupling agent (methacryloxypropyltrimethoxy silane). The filler particles were graded in size and were either spherical or irregular. The spherical particles were silica and made from solution (SiO2), the irregular particles were ground glass melts (Ba-Al-B-silicate glass). The composition of the resin composites is summarized in Table 1.

### 2.1. Specimen Preparation

Four disc specimens (10 mm × 2 mm) were prepared for each material used. Teflon moulds were used to prepare these specimens. The samples were irradiated for 40 s from each surface with a light-curing unit (Optilux 501, Demetron, Danbury, USA) emitting 550 mW/cm2 irradiance, as measured with the radiometer incorporated into the appliance. After polymerization, all specimens were polished.

### 2.2. Polishing Procedures

The samples were initially finished with a sequence of 400-, 600-, 800- and 1200-grit SiC papers under continuous water cooling. To obtain a glossy surface, the specimens were further polished with Sof-Lex contouring and polishing discs (3M Dental Products, St. Paul, MN, USA). The discs were used at medium speed approximately 10,000 rpm for 15 seconds. The finishing and polishing procedures were carried out by one operator to minimise the variability. Finally, the specimens were placed in an ultrasonic water bath (Transonic T 310, Camlab Limited, Cambridge, England) for 2 min to remove any residual debris. The specimens were then stored in distilled water at 37°C for 24 h.

### 2.3. Surface Gloss

The surface gloss of each sample was measured with a glossmeter (Novo Curve, Rhopoint, Instrumentation LTD, East Sussex, England) which was calibrated against a black glass standard provided by the manufacturer. Five measurements per specimen were performed at 60° light incidence, and a mean value for each measured specimen was chosen. These measurements were taken at baseline and after brushing.

### 2.4. Surface Roughness

Surface roughness for all the samples was measured with a noncontact single point sensor: Talysurf CLI 1000 (Ametek Taylor Hobson Precision, Leicester, UK). Each sample was placed over a flat surface above the cross-slides and scanned by a confocal optical single point sensor (CLA 3 mm) with 0.25 mm cutoff length. The sampling rate of the gauge was 500 Hz. The mode of measurement was East-West gauge measurement direction, i.e., from right to left rather than from top to bottom. For each sample, the start and end of scan points were adjusted with a maximum spacing of 10 *μ*m. The measurement speeds were 5 mm/s and 5 mm/s on return. The data obtained as a result of surface scanning were then analysed by TalyMap (Ametek Taylor Hobson Precision, Leicester, UK) analysis software to provide 2D and 3D surface profiles and calculate surface roughness parameters and create a top 3D view. The following 2D roughness parameters were measured: [1] Ra (the arithmetic mean of the absolute departures of the roughness profile from the mean line) [2] and Rt (the maximum peak to valley height of the profile in the assessment length), and 3D roughness parameters were [3] Sa (the arithmetic mean deviation of the surface) [4] and St (the total height of the surface, the height between the highest peak and the deepest valley).

**Figure 1:**
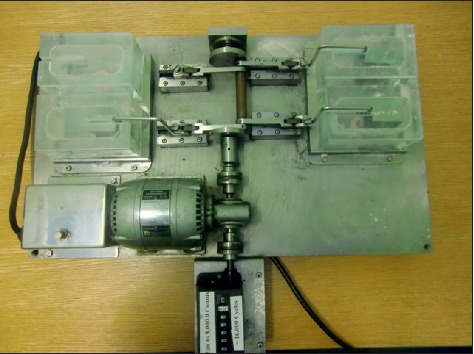
Toothbrush-simulating machine.

**Figure 2:**
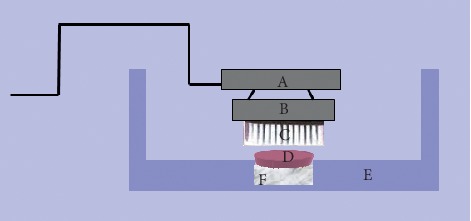
Schematic diagram of tooth-brushing abrasion apparatus. (A) 2.5N metal load, (B) toothbrush holder, (C) toothbrush head, (D) composite sample, (E) glass container, and (F) silicon mould.

All samples were then subjected to simulated wear in a custom-built “tooth-brushing machine” which has been described previously [7]. The toothbrush machine had four separate stations and four separate toothbrush holders which were driven by a motor (Figure 1). Therefore, four specimens were simultaneously but individually subjected to an equal amount of toothbrush/toothpaste abrasion during each testing period. Each toothbrush (Oral-B 40 Indicator, regular), was fixed in the toothbrush holder so that all the bristles were in contact with the specimen (Figure 2). The testing machine was adjusted to apply 2.5 N vertical load on the specimen during horizontal movement of the toothbrush throughout the test. A commercial tooth paste (Colgate Total, Colgate-Palmolive, Guildford, UK) was used to form a slurry according to ISO/TS 1469-1 (2 : 1, water : toothpaste). All specimens were brushed for 20,000 cycles. This corresponds to approximately 4 years of tooth brushing [13]. 2D and 3D roughness parameters were measured after 10,000 cycles and after 20,000 cycles of tooth brushing.

All data were entered in a statistical software package (SPSS ver.16.0, Chicago, IL, USA) and evaluated using one-way analysis of variance (ANOVA) and Bonferroni post hoc test (*p* < 0.05) for the difference between surface gloss (at baseline and after 20,000 cycles of tooth-brushing abrasion) and for the difference between surface roughness (at baseline, after 10,000 cycles and after 20,000 cycles). Linear correlation was checked between filler size and each roughness parameter (at baseline, after 10,000 cycles and after 20,000 cycles).

## 3. Results

### 3.1. Gloss Retention

**Figure 3:**
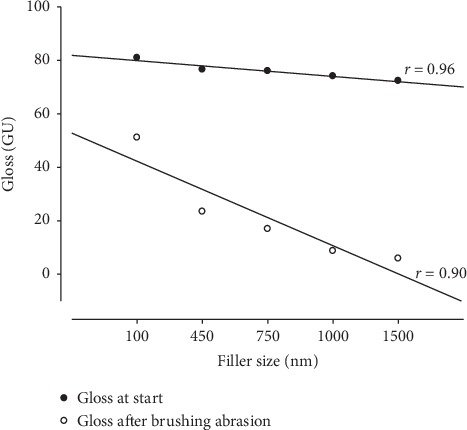
Linear correlation between filler size and gloss retention.

Gloss values ranged between 72.3 and 84.3 GU before abrasion and between 5.9 and 61.3 GU after toothbrush abrasion (Table 2). For all materials, a statistically significant reduction in gloss was observed after toothbrush abrasion (*p* < 0.05). I4 (1500 nm) exhibited the lowest gloss retention (8.1 %) before and after tooth-brushing abrasion. Nonetheless, at baseline, it was not significantly different from I6 (multimodal distribution material 450, 700 and 1500 nm). The highest gloss retention (72.8%) was shown by TC (40, 200 and 1000 nm), and it was significantly different from the other composites. Surface gloss values were strongly correlated with filler size before (*r* = 0.96) and after tooth-brushing abrasion (*r* = 0.90) (Figure 3).

### 3.2. Surface Roughness

All materials exhibited very smooth surfaces before toothbrush abrasion. Initial values ranged from 0.01–0.03 *μ*m (Ra), 0.27–0.35 *μ*m (Rt), and 0.11–0.57 *μ*m (Sa) and 31.94–80.63 *μ*m (St). After 10,000 cycles of abrasion values ranged from 0.08–2.04 *μ*m (Ra), 1.14–2.60 *μ*m (Rt), and 0.61–2.03 *μ*m (Sa) and 40.62–91.92 *μ*m (St). After 20,000 cycles 0.71–3.35 *μ*m (Ra), 1.90–3.11 *μ*m (Rt), 1.17–2.93 *μ*m (Sa), and 50.64–99.82 *μ*m (St). All 2D and 3D surface roughness measurements are summarized in Table 3 and 4, respectively.

Bonferroni post hoc comparisons revealed significant mean differences in Ra, Rt, Sa, and St values before and after toothbrush abrasion. These differences were more prominent for the unimodal larger filler size materials (750, 1000, and 1500 nm) compared to smaller filler size materials (100–450 nm) regardless the filler shape.

**Figure 4:**
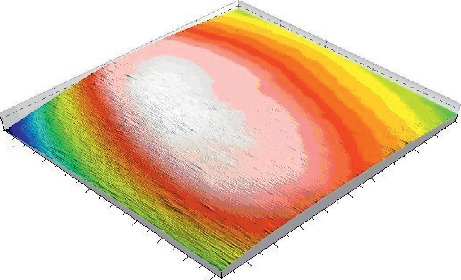
3D model of the scanned sample.

**Figure 5:**
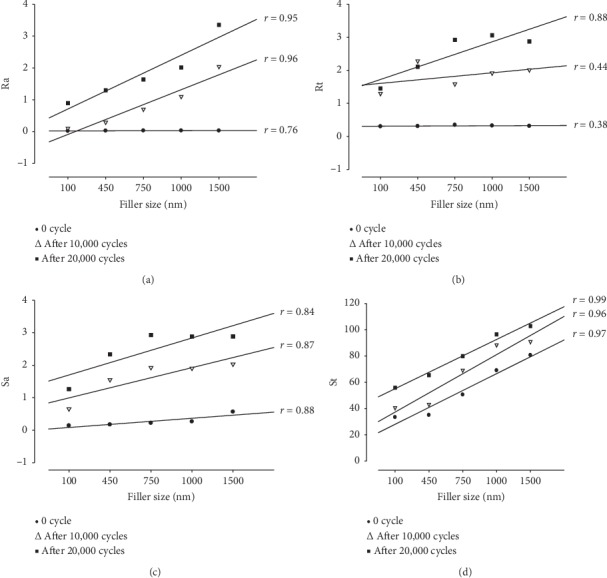
Linear correlation between filler size and surface roughness parameter.

Among the multimodal composite resins, TC exhibited the lowest values of both 2D and 3D data measurements before and after brushing; moreover, this material, exhibited the lowest value among all materials retested in this study. Additionally 3D model was created (Figure 4). Possible correlations between roughness parameters and filler size were investigated at baseline (after polishing), after toothbrush abrasion (10,000 cycles) and after toothbrush abrasion (20,000 cycles). These are shown in Figure 5. Correlation values ranged from (*r* = 0.99) for St after toothbrush abrasion (20,000 cycles) to (*r* = 0.38) for Rt at the baseline.

## 4. Discussion

The quality of a resin composite restoration surface depends on two main factors which are the material composition and the polishing system used. Previous studies have shown that the polishing system not only influences surface roughness, gloss, and colour stability but may also have a role in other properties such as microhardness and microleakage [11, 14, 15].

The wear of resin composite material starts with gradual removal of the organic component which leads to projection of unsupported filler particles and subsequent exfoliation [16]. Thus, the interparticle space has been shown to play an important role in the wear resistance of resin composites, as the interparticle space reducing the wear resistance of composite material improves. This can be explained since in fillers that are closer together the organic resin is more protected from abrasives, and thus the wear is reduced [17].

Gloss and surface roughness are usually linked together, and the relationship between the two has been illustrated in previous studies [14, 18]. One can affect the other, and it is beneficial to study them simultaneously to obtain a more representative view of the behavior of material in terms of surface properties. The method used in this study to evaluate surface roughness is relatively novel and differs from the conventional methods used in the majority of previous studies [7]. It has several advantages which are: noninvasive as it is scanned by a confocal optical single point sensor rather than using a stylus that touches the sample and can obtain parameters not only in 2D but also in 3D. 3D mapping is more representative of the surface and thus leads to more reliable results because it is defined in one sampling area and can generate 3D model, unlike 2D measurements which rely on multiple lengths of the sample [19, 20]. This difference could be an explanation of the conflicting significance differences in amplitude roughness parameter values at 0 cycle. 2Ds parameters showed no significant difference between materials tested at 0 cycle (Table 3), whereas 3Ds parameters exhibited significant difference at 0 cycle (Table 4). Despite all these advantages, this method is relying on an experienced operator and can be more time consuming. Moreover, 2D parameters still useful guidelines which help to proper understanding of surface roughness values and make the comparisons with other studies easier [19].

For all materials tested, the surface became statistically less glossy after toothbrush abrasion, and this was statistically correlated to filler size. A clear trend could be seen where an increase in filler size led to reduction in gloss before and after brushing abrasion (Figure 3). Thus, the first null hypothesis was rejected. This is in agreement with previous studies [7, 21]. However, the correlation between filler size and surface gloss is stronger in the current study. Among the multimodal resin composites, TC revealed higher gloss than any other material used in the study, whether multimodal or unimodal.

Toothbrush abrasion increased all roughness parameters tested both in 2D and 3D measurements. The difference between materials was statistically significant (*p* < 0.05), and thus the second hypothesis was rejected. There was a strong correlation between filler size and Ra, Sa, and St (Figure 5). However, for Rt, the correlation became more pronounced after 20,000 cycles of toothbrush abrasion which corresponds to 4 years of tooth brushing.

The unimodal resin composites which have larger filler sizes I4 (1500 nm) and I3 (1000 nm) exhibited the highest values of all 3D roughness parameters and Ra parameter in 2D measurements before and after tooth abrasion. This was more prominent in 3D roughness parameters. This result is in conflict with other published results [7, 21]. This could be due to the difference in the technique used to evaluate surface roughness (2D and 3D) and also could be due to larger variations in filler sizes used in this study that might illustrate differences more clearly.

## 5. Conclusions

In this study, filler size was shown to have a significant influence on both surface properties examined. The effect was illustrated more clearly in terms of retention. After toothbrush abrasion that simulated long-term clinical service, the resin composites with the smaller filler size demonstrated the highest retention values. This also highlights the importance of simulation experiments that will discriminate between materials more accurately. Despite few differences being observed for gloss and roughness after polishing, more could be seen after the abrasion process. This sets a limitation in reporting only those initial values since it can lead to misleading information for practitioners expecting that two materials will perform the same.

## Figures and Tables

**Table 1:** Composition of materials used in the study.

Resin composite	Filler particles (ground glass (Ba-Al-B-silicate glass))				Matrix
	Shape	Size (nm)	Wt (%)	Vol (%)	
I1	Irregular	450	76.4	56.7	Bis-GMA, UDMA, and TEGDMA
I2	Irregular	700	76.4	56.7	
I3	Irregular	1000	76.4	56.7	
I4	Irregular	1500	76.4	56.7	
I5	Irregular	450, 1000 (1 : 3)	76.4	56.7	
I6	Irregular	450, 700, and 1500 (1 : 1:3)	76.4	56.7	
SP	Spherical	100	72.4	56.7	
Tetric ceram (TC)					
Lot: C49490	Irregular & spherical	40, 200, and 1000	79	60	

**Table 2:** Mean (SD) of gloss of all material tested before and after tooth-brushing abrasion of 20,000 cycles.

Material	Gloss (initial)	Gloss (after tooth-brushing abrasion)
Sp	80.90 (0.70)^a^*∗*^^	51.10 (0.44)^a^*∗*^^
I1	76.50 (0.52)^b^*∗*^^	23.40 (0.39)^b^*∗*^^
I2	75.95 (0.52)^b,e^*∗*^^	16.90 (0.48)^c^*∗*^^
I3	74.03 (0.22)^c^*∗*^^	8.68 (0.63)^d^*∗*^^
I4	72.30 (0.29)^d^*∗*^^	5.85 (0.33)^e^*∗*^^
I5	75.18 (0.34)^e^*∗*^^	14.70 (0.56)^f^*∗*^^
I6	72.73 (0.46)^d^*∗*^^	11.98 (0.59)^g^*∗*^^
TC	84.30 (0.47)^f^*∗*^^	61.33 (1.10)^h^*∗*^^

Within each column, similar superscripts indicate no significant difference (*p* < 0.05). Within each group, ^*∗*^ represent significant differences before and after tooth-brushing abrasion.

**Table 3:** Mean (SD) of 2D roughness parameters.

Group	Tooth-brushing abrasion					
	At start (0 cycle)		After 2 years brushing (after 10,000 cycles)		After 4 years brushing (after 20,000 cycles)	
	Ra	Rt	Ra	Rt	Ra	Rt
Sp	0.02 (0.01)^a^*∗*^^	0.30 (0.04)^a^*∗*^^	0.10 (0.01)^a,b^*∗*^^	1.30 (0.02)^a^*∗*^^	0.90 (0.05)^a^*∗*^^	1.90 (0.05)^a^*∗*^^
I1	0.03 (0.01)^a^*∗*^^	0.31 (0.02)^a^*∗*^^	0.30 (0.06)^a^*∗*^^	2.27 (0.24)^b^*∗*^^	1.30 (0.07)^b^*∗*^^	2.94 (0.06)^b^*∗*^^
I2	0.03 (0.09)^a^*∗*^^	0.35 (0.05)^a^*∗*^^	0.70 (0.03)^c^*∗*^^	2.28 (0.12)^b,c^*∗*^^	1.64 (0.05)^c,f^*∗*^^	3.02 (0.14)^b^*∗*^^
I3	0.03 (0.01)^a^*∗*^^	0.33 (0.02)^a^*∗*^^	1.10 (0.08)^d^*∗*^^	2.59 (0.07)^c^*∗*^^	2.01 (0.07)^d^*∗*^^	3.06 (0.06)^b^*∗*^^
I4	0.03 (0.01)^a^*∗*^^	0.31 (0.04)^a^*∗*^^	2.04 (0.15)^e^*∗*^^	2.60 (0.21)^c^*∗*^^	3.35 (0.31)^e^*∗*^^	3.10 (0.12)^b^*∗*^^
I5	0.03 (0.01)^a^*∗*^^	0.32 (0.03)^a^*∗*^^	0.48 (0.12)^a,b,c^*∗*^^	2.03 (0.04)^b^*∗*^^	1.38 (0.11)^b,c^*∗*^^	2.69 (0.06)^c^*∗*^^
I6	0.03 (0.01)^a^*∗*^^	0.32 (0.03)^a^*∗*^^	0.64 (0.04)^c^*∗*^^	2.09 (0.02)^b^*∗*^^	1.74 (0.08)^f^*∗*^^	3.11 (0.06)^b^*∗*^^
TC	0.01 (0.01)^a^*∗*^^	0.27 (0.02)^a^*∗*^^	0.08 (0.02)^a^*∗*^^	1.14 (0.04)^a^*∗*^^	0.71 (0.04)^g^*∗*^^	2.07 (0.08)^a^*∗*^^

Within each column, similar superscripts indicate no significant difference (*p* < 0.05). Within each group, ^*∗*^ represent significant differences in Ra and Rt among tooth-brushing abrasion cycles.

**Table 4:** Mean (SD) of 3D roughness parameters.

Group	Tooth-brushing abrasion					
	At start (0 cycle)		After 2 years brushing (after 10,000 cycles)		After 4 years brushing (after 20,000 cycles)	
	Sa	St	Sa	St	Sa	St
Sp	0.14 (0.04)^a^*∗*^^	33.35 (2.10)^a^*∗*^^	0.66 (0.05)^a^*∗*^^	40.62 (0.70)^a^*∗*^^	1.26 (0.09)^a^*∗*^^	55.87 (0.63)^a^*∗*^^
I1	0.22 (0.02)^a,b^*∗*^^	35.02 (2.33)^a^*∗*^^	1.55 (0.07)^b^*∗*^^	43.16 (1.90)^a,b^*∗*^^	2.33 (0.21)^b^*∗*^^	65.40 (0.42)^b^*∗*^^
I2	0.17 (0.03)^b,c^*∗*^^	50.52 (1.89)^b^*∗*^^	1.93 (0.08)^c^*∗*^^	69.19 (0.79)^c^*∗*^^	2.93 (0.14)^c^*∗*^^	79.90 (0.48)^c^*∗*^^
I3	0.27 (0.03)^c,d^*∗*^^	69.00 (2.51)^c^*∗*^^	1.91 (0.04)^c^*∗*^^	88.45 (1.56)^d^*∗*^^	2.88 (0.12)^c^*∗*^^	96.52 (0.65)^d^*∗*^^
I4	0.57 (0.04)^e^*∗*^^	80.63 (2.15)^d^*∗*^^	2.03 (0.09)^c^*∗*^^	90.92 (1.18)^d^*∗*^^	2.89 (0.08)^c^*∗*^^	102.82 (0.23)^e^*∗*^^
I5	0.30 (0.02)^d^*∗*^^	42.08 (1.82)^e^*∗*^^	1.60 (0.08)^b^*∗*^^	55.76 (0.43)^e^*∗*^^	1.55 (0.05)^d^*∗*^^	86.60 (0.63)^f^*∗*^^
I6	0.30 (0.03)^d^*∗*^^	51.97 (3.02)^b^*∗*^^	1.53 (0.04)^b^*∗*^^	46.25 (0.63)^b^*∗*^^	1.63 (0.09)^d^*∗*^^	82.70 (0.47)^g^*∗*^^
TC	0.11 (0.02)^a^*∗*^^	31.94 (2.38)^a^*∗*^^	0.61 (0.04)^a^*∗*^^	40.94 (0.98)^a^*∗*^^	1.17 (0.04)^a^*∗*^^	50.64 (0.83)^h^*∗*^^

Within each column, similar superscripts indicate no significant difference (*p* < 0.05). Within each group, ^*∗*^ represent significant differences in Sa and St among tooth-brushing abrasion cycles.

## Data Availability

The data used to support the findings of this study are available from the corresponding author upon request.
